# Detection of magnetic tracers with M_x_ atomic magnetometer for application to blood velocimetry

**DOI:** 10.1038/s41598-021-86358-0

**Published:** 2021-03-30

**Authors:** Asieh Soheilian, Mohammad Mehdi Tehranchi, Maliheh Ranjbaran

**Affiliations:** 1 Laser and Plasma Research Institute, Shahid Beheshti University, Tehran, Iran; 2 Physics Department, Shahid Beheshti University, Tehran, Iran; 3 Department of Physics, Central Tehran Branch, Islamic Azad University, Tehran, Iran

**Keywords:** Biophysics, Optics and photonics

## Abstract

In the new generation of blood velocimeter systems, considerable attention has been paid to atomic magnetometers due to their high resolution and high sensitivity for detection of magnetic tracers. Passing the magnetic tracers adjacent to the atomic magnetometer produces a spike-like signal, the shape of which depends on the position of the tracer, as well as its velocity and orientation. The present study aimed to evaluate the effect of abrupt variations in the instantaneous velocity of the magnetic tracer on the magnetometer response compare to constant velocity. Modeling the magnetic tracer as a dipole moment indicated that the velocity dependence of the magnetic field and local magnetic field gradient associated with moving magnetic tracer cause the spike-like signal to go out of symmetry in the case of variable velocity. Based on the experimental results, any instantaneous variation in tracer velocity leads to shrinkage in the signal width. The behavior has been studied for both magnetic microwire with variable instantaneous velocity and magnetic droplets in stenosis artery phantom. In addition, the position of the tracer could be detected by following the shrinkage behavior which may occur on the peak, valley, or both. These advantageous outcomes can be applied for high sensitivity diagnosis of arterial stenosis.

## Introduction

Targeted treatment strategies have attracted a lot of attention to improve therapeutic and diagnostic accuracy studies^1,2^, which aimed at reducing toxic side effects of drugs on healthy tissues in drug delivery^3^, minimizing the invasiveness of surgeries^4^, actuating prostheses and implants wirelessly^5^, and localizing the capabilities of the medical imaging precisely^6–8^. Such purposes may be feasible using appropriate nano-to micro-scale materials^9^. Magnetic materials are highly considered in targeted diagnosis and therapy. These materials can be manipulated using external magnetic fields, and their small size presents deep tissue penetration which is an important parameter in medical diagnosis such as vascular imaging^10–17^. Vascular imaging can be used to characterize blood flow for diagnosis of circulatory lesions such as blood clots and blockages in the arteries and veins^18^. To detect these lesions several vascular imaging modalities are used widely such as laser Doppler imaging and ultrasound Doppler imaging that employ Doppler effects to evaluate blood flow^19,20^. Moreover, today's new imaging methods based on magnetic measurements are being developed that made them usable for vascular imaging in more depth than most conventional optical and doppler velocimeters^21^. These medical imaging modality systems are tracer-based which require sensitive detection methods for detecting weak magnetic fields.

Recently, several detection methods have been developed in biological imaging such as superconducting quantum interference devices (SQUIDs) for monitoring of blood flow using a magnetic microparticle^22^, giant magnetic impedance (GMI) sensors for in-flow detection of superparamagnetic iron oxide nanoparticles in order to ex-vivo analysis of blood flow^23^, diamond-based magnetometers for detecting a single super-paramagnetic nanoparticle capable of molecular binding event detection^24^, and atomic magnetometers for subpicomolar detection of magnetic nanoparticles in a flowing media (water and blood)^25^ which indicated a comparative high sensitivity. Some requirements such as size, price, and operating consideration make one of these magnetometers, which is preferred for application-specific usage. In the present study, an atomic magnetometer which can operate without requiring cryogenics and offer the highest sensitivity at low frequencies was used^21^. The low frequency of the magnetometer allows the excitation frequency of the drive field to be lowered which reduces concerns regarding heating and nerve stimulation of peripheral tissues^26^. Despite these advantages, the application of this magnetometer in biomedical imaging is in its infancy, which requires some problems to overcome before being applied clinically in humans.

The extraction of detection information from the atomic magnetometer signal is considered as one of the less explored problems in magnetic target detection with the atomic magnetometer. The information includes magnetization, position, velocity, and orientation of the magnetic target. Xu et al. first reported that a spike-like signal is produced when the magnetic particle at a water flow passes the magnetometer. In addition, the detection limit of the signal relied on the flow rate and particle magnetization^27^. Then, Maser employed a microfabricated SERF magnetometer and improved the sensitivity of the particle detection, the results of which showed that the orientation of the particle can change the shape of the signal^28^. Recently, the manner of the signal dependency on the magnetism, velocity, and orientation of the particle was evaluated. Based on the results, the spike-like signal is produced by simultaneous changing the magnetic field and the local magnetic field gradient of the moving magnetic target. Magnetic target with stronger magnetization produces a stronger signal and the orientation of the velocity can inverse the amplitude of the signal^29^. However, the dependency of the signal to instantaneous velocity was ambiguous.

In a stenosed artery, the blood velocity changes instantaneously. By detecting instantaneous changes in blood velocity, the location of the blockage can be detected. The present study sought to diagnose the blockage in an artificial arterial vessel by tracking changes in the velocity of the magnetic fluid which are recorded with an atomic magnetometer. To understand the functional characteristics of an atomic magnetometer in response to instantaneous velocity, we have used a magnetic microwire with high permeability. Besides, the magnetic microwire was simulated as a dipole moment and the dependence of its magnetic field and local magnetic field gradient to instantaneous velocity was evaluated. Moreover, to become closer to a biological situation, a stenosed arterial phantom was considered. In this phantom, instantaneous velocity increases abruptly in the vessels containing stenosis. Further, the effect of these abrupt velocity changes on the shape of output spike-like signal was thoroughly studied. The results of the present study can open new research opportunities in blood velocimeter systems which provide early detection of the stenosis to prevent from possible heart attacks and strokes.

## Experimental setups

### Experimental setup for detection of magnetic microwire

**Figure 1:**
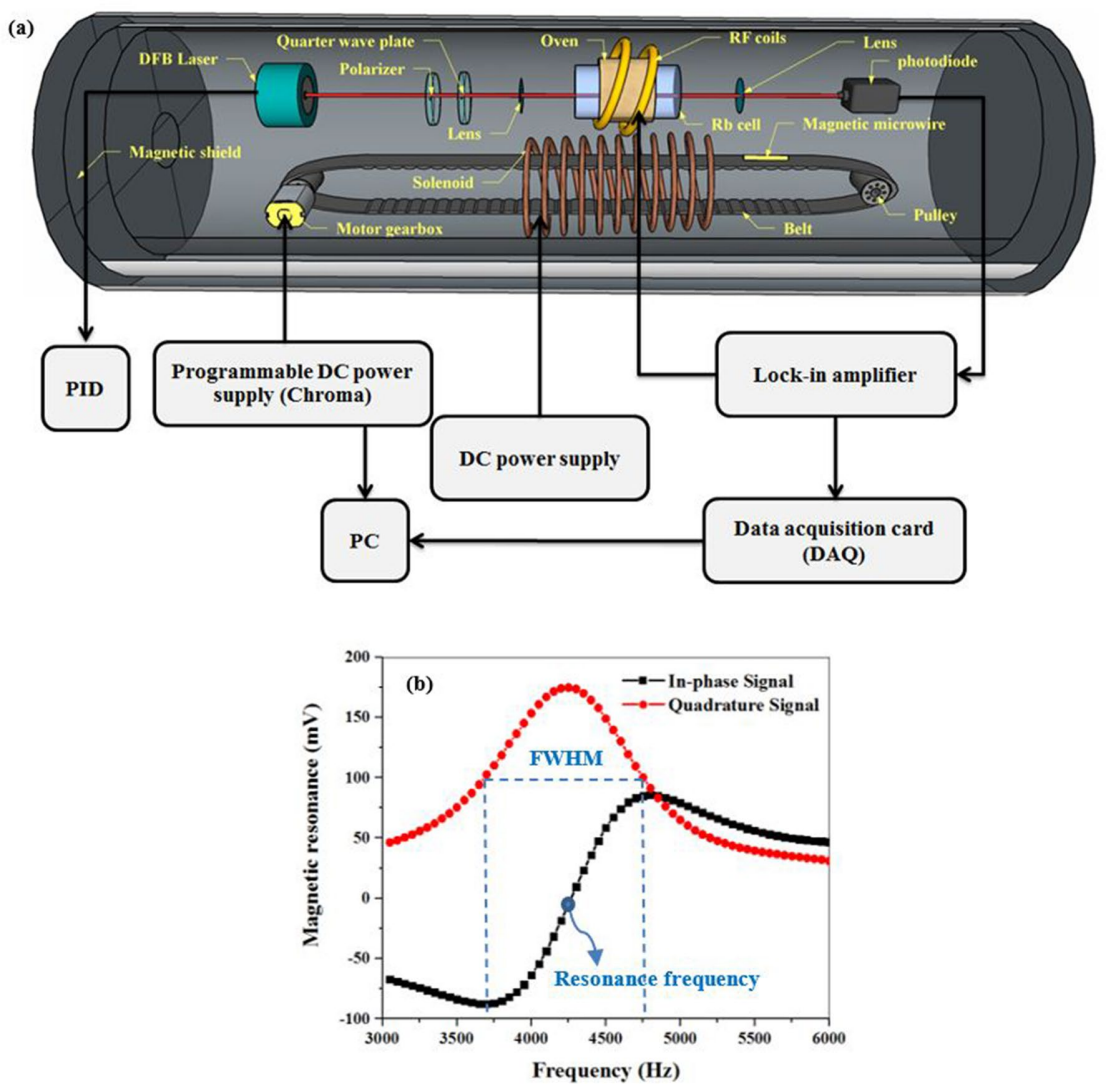
(**a**) Experimental setup for detecting the magnetic microwire, and (**b**) magnetometric signal as the output of the phase-sensitive detection method.

Before tracking magnetic fluid through an arterial phantom and diagnosis the stenosis location, it is necessary to identify functional properties of the atomic magnetometer in magnetic velocimetry. Thus, we desired a small ferromagnetic material with a pronounced magnetic response. Amorphous and nanocrystalline wires are the best candidates which present rectangular hysteresis loops with soft magnetic properties. So, an amorphous co-based (Co_68.15_ Fe_4.35_ Si_12.5_B_15_) microwire of particular dimensions (120 µm diameter and 1 mm length) was used in this study^30,31^. The permeability of the magnetic microwire is in the order of ${10}^{4}$. To stimulate the magnetization of the ferromagnetic microwire, it was subjected to a static magnetic field (4 mT) in a long solenoid with a 2 cm diameter, 36 cm length and 2500 turns. As shown in Fig. 1a, the current of the solenoid is controlled by a DC power supply.

Additionally, the magnetic microwire was placed on a belt stretching around two pulleys in order to generate a variable motion profile. Here, the drive system to build up a variable speed was a motor Gearbox controlled by a programmable DC power supply (Chroma 6104). The output of the power supply was defined via LabVIEW through GPIB interface.

Magnetic detection of this moving magnetic microwire was performed inside a three-layer µ-metal magnetic shielding based on M_x_ configuration of the atomic magnetometer, as displayed in in Fig. 1a^32–39^. This magnetometer was based on optically-detected magnetic resonance in spin-polarized ^85^Rb vapor which was included in an Octadecyltrichlorosilane (OTS) coated Pyrex glass cell filled with 10 torr of N_2_ gas. The sensing volume was defined with the cell dimensions, which was cylindrical with 2.5 cm diameter and 5 cm height. In the next procedure, Rb vapor was generated by heating the cell to around 60 °C.

A single frequency-locked by circularly-polarized beam (to the D_1_ transition: 794.8 nm) created spin polarization in the atomic medium and also probe the magnetization (the laser beam controlled with a loop feedback controller circuit known as a PID circuit). In this way, the medium acquired a non-isotropic distribution of magnetization called “orientation”. The magnetization interacted with the static magnetic field B_0_, the strength of which was described with Larmor precession frequency of ${\upomega }_{\mathrm{L}}={\upgamma }_{\mathrm{F}}\left|{\mathrm{B}}_{0}\right|$ (${\upgamma }_{\mathrm{F}}$ is equal to ground state gyromagnetic ratio). This precession reached a steady-state magnetization under the influence of external magnetic fields and relaxation mechanisms. A weak oscillating magnetic field B_rf_(t) mentioned this precession coherently which was generated by a lock-in amplifier oscillator (Stanford Research Systems, SR830).

The evolution of spin polarization under the influence of the static and time-dependent magnetic fields was detected as the transmitted light intensity variates on a photodetector (2031 New Focus). This magnetometric data were extracted by phase-sensitive detection by using the lock-in amplifier signal which interfaced with a PC using a data-acquisition card (DAC). When the radio frequency was tuned near the Larmor frequency (induced by the external magnetic field), a resonance behavior was observed in the in-phase and quadrature components of the magnetometric signal (magnetic resonance signal) which have dispersive and absorptive Lorentzian dependence, respectively (Fig. 1b). Finally, the entire process of the velocity control and data acquisition was monitored and supervised by a LabVIEW program.

To detect magnetic target, the magnetometer signal was locked in the near resonance point where the dependency of the in-phase signal is linear. The resonance frequency is related to the stray magnetic field (B_0_) of the solenoid on the cell location that was calculated as 4250 Hz. The sensitivity of the atomic magnetometer was obtained in the order of pT by measuring its noise spectral density^29^. Using the phase-locked loop, we measured any variation of light intensity associated with the deviation of the frequency δf and resonance curve slope which were proportional to the magnetic field and local magnetic field gradient of the moving magnetic target, respectively^29^.

### Experimental setup for detection of magnetic droplets

**Figure 2:**
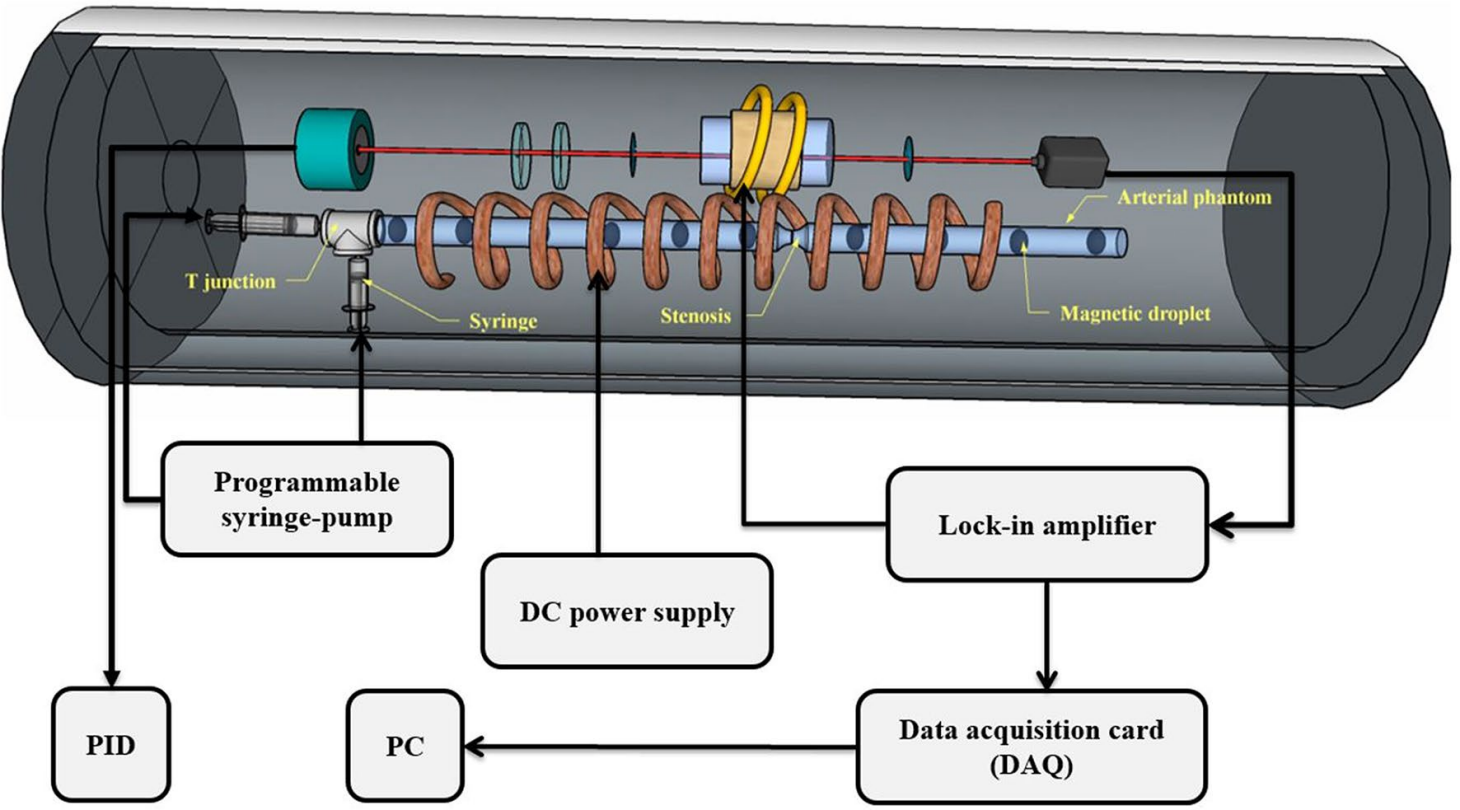
Experimental setup for detecting the magnetic droplets.

At the next step, to elucidate the role of instantaneous velocity measurements in stenosis detection, we have fabricated two artificial stenosis in an arterial phantom with different length of 7 mm and 14 mm. The phantom was a silicone tube with a 3 mm inner diameter. Small droplets of magnetic nanoparticle (Resovist (Bayer Schering Pharma) with concentration of 0.5 mmol Fe/ml) with equivalent diameter (1 mm) were generated in the tube using a programmable syringe-pump (IMSP96-1) and T-shaped inlet junction (As shown in Fig. 2). Magnetic nanoparticle droplets separated by the continuous air injection when we applied 20 mm/h flow rate for magnetic fluid phase and 600 mm/h flow rate for air phase. So, Liquid droplets generated within a gaseous flow (liquid-in-gas systems)^40^. The velocity of magnetic droplets is constant and slow. Therefore, the shape of the magnetic droplets doesn’t change and they don’t rotate during their movement.

Magnetic droplets were magnetized with a long solenoid (with a 1 cm diameter and 36 cm length). In the weak magnetic field of the order of a few mT, the relation between the magnetism of the nanoparticles immersed in the droplets and applied magnetic field is linear^41^. So, the following relation can be attributed to the magnetism,

1 $$\mathrm{M}={\upchi }_{\mathrm{m}}{\mathrm{CV}}_{\mathrm{t}}{\mathrm{H}}_{\mathrm{ext}},$$

where ${\upchi }_{\mathrm{m}}$ is the molar magnetic susceptibility, C is the concentration of the MNPs, and ${\mathrm{V}}_{\mathrm{t}}$ is the volume of the droplets. So, we can attribute a net magnetization to the magnetic droplets that the expression of the magnetic field created at the sensor location equals to^42^,

2 $${\overrightarrow{\mathrm{B}}}_{\mathrm{np}}=\frac{{\upmu }_{0}}{4\uppi }\overrightarrow{\mathrm{grad}}\left(\frac{\overrightarrow{\mathrm{M}}.\overrightarrow{\mathrm{r}}}{{\mathrm{r}}^{3}}\right) .$$

To detect the magnetic droplets, the same as previous experimental setup section, the magnetometer signal was locked on the resonance frequency of the stray magnetic field (B_0_) of the solenoid and the variation of light intensity was recorded.

## Results and discussion

### Formation of the spike-like signal at constant velocity

**Figure 3:**
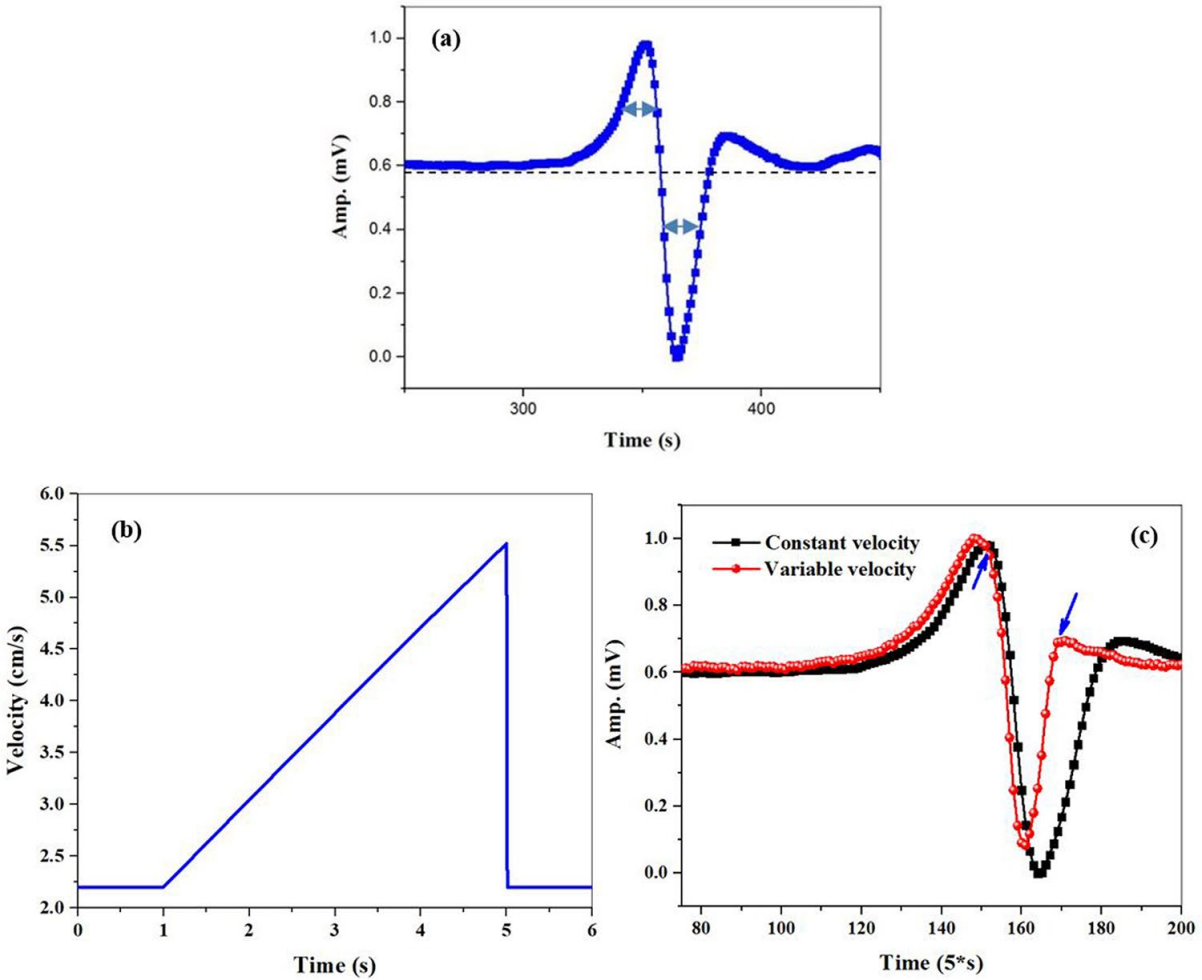
(**a**) The spike-like signal related to moving microwire with constant velocity. (**b**) Triangular variable velocity profile, (**c**) the spike-like signals related to the moving microwire with constant (black squares) and variable velocity (red circles) (time interval of applying instantaneous velocity marked with blue arrows).

As reported in our previous paper, we utilized an atomic magnetometer for detecting a moving magnetic microwire. While the working frequency of the magnetometer was locked to the Larmor resonance frequency, a spike-like signal was detected with each passing the magnetic microwire with a constant velocity of 2.7 cm/s. This was while the vertical position of the moving microwire was 4 cm relative to the cell. As illustrated in Fig. 3a, the peak (the valley) of the signal corresponds to the passage of the microwire near the first (second) edge of the Rb vapor cell. To analyze the effective parameters in the formation of this spike-like signal, the parameters broadening the magnetic resonance signal line-width were studied (Fig. 1b). The full-width of half maximum (FWHM) of the quadrature magnetic resonance signal (which equals to the peak-to-valley frequency interval of the in-phase magnetic resonance signal) is described by

3 $$\Gamma =\frac{1}{{\mathrm{T}}_{2}}\sqrt{1+{(\upgamma {\mathrm{B}}_{\mathrm{rf}})}^{2}{\mathrm{T}}_{1}{\mathrm{T}}_{2}},$$

where T_1_ and T_2_ are the longitudinal and transverse relaxation rates, respectively, and B_rf_ is considered as the amplitude of the oscillating magnetic field^37,43,44^. As it was already mentioned, T_1_ and B_rf_ (equals to 10 nT) are invariant when the moving magnetic microwire passes the atomic magnetometer. Therefore, the changes in the FWHM of the magnetic resonance curve depends only on the transverse relaxation rate variations based on the following proportional equation

4 $$\mathrm{\Gamma \alpha }1/{\mathrm{T}}_{2},$$

where T_2_ is given by

5 $$\frac{1}{{\mathrm{T}}_{2}}=\frac{1}{{\mathrm{T}}_{1}}+\frac{1}{{\mathrm{q}}_{\mathrm{SE}}}{\mathrm{R}}_{\mathrm{SE}}+{\mathrm{R}}_{\mathrm{gr}},$$

where R_SE_ indicates the rate of the spin-exchange collisions which cause dephasing in the precessed atoms coherently. In addition, q_SE_ shows the spin-exchange broadening factor and R_gr_ is considered as the effect of magnetic field gradient which disturbs the spin coherency and can affect the width of the magnetic resonance curve^44–50^.

In general, the displacement of the magnetic microwire changes the magnetic field and local magnetic field gradient, simultaneously. Changes in the local magnetic field gradient in different positions can change the width of the magnetic resonance curve, $\Gamma$ (or resonance curve slope (m)). On the other hand, locating the microwire in different positions leads to change of the magnetic field and deviate the system Larmor frequency, the effect of which appeared in changing the turning point frequency (resonance frequency of spins (dF)) of the magnetic resonance curve. Simultaneous changes of the magnetic field and local magnetic field gradient in different locations can alter the light intensity which resulted in the spike-like signal^31^. As shown in the signal in Fig. 3a, the motion of microwire with constant velocity produces a spike-like signal with a nearly symmetrical shape, where the width of the peak and valley is nearly identical.

### Formation of the spike-like signal in abrupt velocity changes

The response of the atomic magnetometer was studied in tracking a magnetic microwire with constant velocity. In this section, a magnetic microwire with variable instantaneous velocity was tracked. The results can be used to study blood velocimetry.

To this aim, a microwire was placed on the belt while its velocity was controlled with a gearbox motor. First, the gearbox motor was driven with a uniform velocity. Accordingly, a triangular voltage was applied to the motor gearbox terminals in order to simulate an abrupt velocity change in the microwire motion.

Figure 3b displays the triangular voltage profile applied to the motor gearbox terminals. A spike-like signal was detected by recording the changes of light intensity because of passing the microwire (Fig. 3c). In addition, it presents the comparison of the moving microwire with constant velocity (black squares) and an abrupt change in the velocity (red circles).

Regarding the comparison between the curves with red circles and black squares (Fig. 3c), it is suggested that the width of the spike-like signal decrease when an instantaneous variation is exerted in the microwire velocity. So, this qualitatively obtained result shows that this magnetometer is sensitive to instantaneous changes in velocity.

Since the spike-like signal is obtained from the simultaneous changes of the magnetic field and the local magnetic field gradient, the dependence of these two components on the variable velocity is obtained. First, a physical insight is required for how the magnetic field and local magnetic field gradient of the tracer depend on its distance from the atomic magnetometer.

### Simulation of the magnetic microwire as a magnetic dipole

The magnetic microwire can be approximately considered as a magnetic dipole moment because the distance from the magnetometer is large compared with the characteristic length of this object. The magnetic field of the dipole can be derived from Maxwell’s equations as follows:

6 $$\mathrm{B}\left(\mathrm{r}\right)=\frac{{\upmu }_{0}}{4\uppi }\frac{3\left(\overrightarrow{\mathrm{m}}.\widehat{\mathrm{n}}\right)\widehat{\mathrm{n}}-\overrightarrow{\mathrm{m}}}{{\mathrm{r}}^{3}},$$

where $\overrightarrow{\mathrm{m}}$ indicates the magnetic moment of the dipole and $\mathrm{r}$ shows its relative position to the magnetometer with direction of $\widehat{\mathrm{n}}=(\frac{\overrightarrow{\mathrm{r}}}{\mathrm{r}})$. The magnetic field gradient is a tensor which is the spatial derivative of the magnetic field of B and is defined as follows:

7 $$\mathrm{G}=\left(\begin{array}{c}\frac{\partial }{\partial \mathrm{x}}\\ \frac{\partial }{\partial \mathrm{y}}\\ \frac{\partial }{\partial \mathrm{z}}\end{array}\right)\left(\begin{array}{ccc}{\mathrm{B}}_{\mathrm{x}}& {\mathrm{B}}_{\mathrm{y}}& {\mathrm{B}}_{\mathrm{z}}\end{array}\right)=\left(\begin{array}{ccc}{\partial }_{\mathrm{x}}{\mathrm{B}}_{\mathrm{x}}& {\partial }_{\mathrm{y}}{\mathrm{B}}_{\mathrm{x}}& {\partial }_{\mathrm{z}}{\mathrm{B}}_{\mathrm{x}}\\ {\partial }_{\mathrm{x}}{\mathrm{B}}_{\mathrm{y}}& {\partial }_{\mathrm{y}}{\mathrm{B}}_{\mathrm{y}}& {\partial }_{\mathrm{z}}{\mathrm{B}}_{\mathrm{y}}\\ {\partial }_{\mathrm{x}}{\mathrm{B}}_{\mathrm{z}}& {\partial }_{\mathrm{y}}{\mathrm{B}}_{\mathrm{z}}& {\partial }_{\mathrm{z}}{\mathrm{B}}_{\mathrm{z}}\end{array}\right).$$

The difference between the magnetic field vectors in two locations can be written by using the gradient tensor as follows:

8 $$\mathrm{dB}=\mathrm{B}-{\mathrm{B}}^{\prime}=\mathrm{Gdr},$$

where ${\mathrm{B}}{^\prime}$ is the magnetic field of the magnetic dipole at the point of ${\mathrm{r}}^{\prime}=\mathrm{r}+\mathrm{n}^{\prime}\mathrm{dr}$ and equals to

9 $${\mathrm{B}}^{\prime}\left(\mathrm{r}\right)=\frac{{\upmu }_{0}}{4\uppi }\frac{3\left(\overrightarrow{\mathrm{m}}.\widehat{{\mathrm{n}}^{\prime}}\right)\widehat{{\mathrm{n}}^{\prime}}-\overrightarrow{\mathrm{m}}}{{\left({\mathrm{r}}^{\prime}\right)}^{3}}.$$

**Figure 4:**
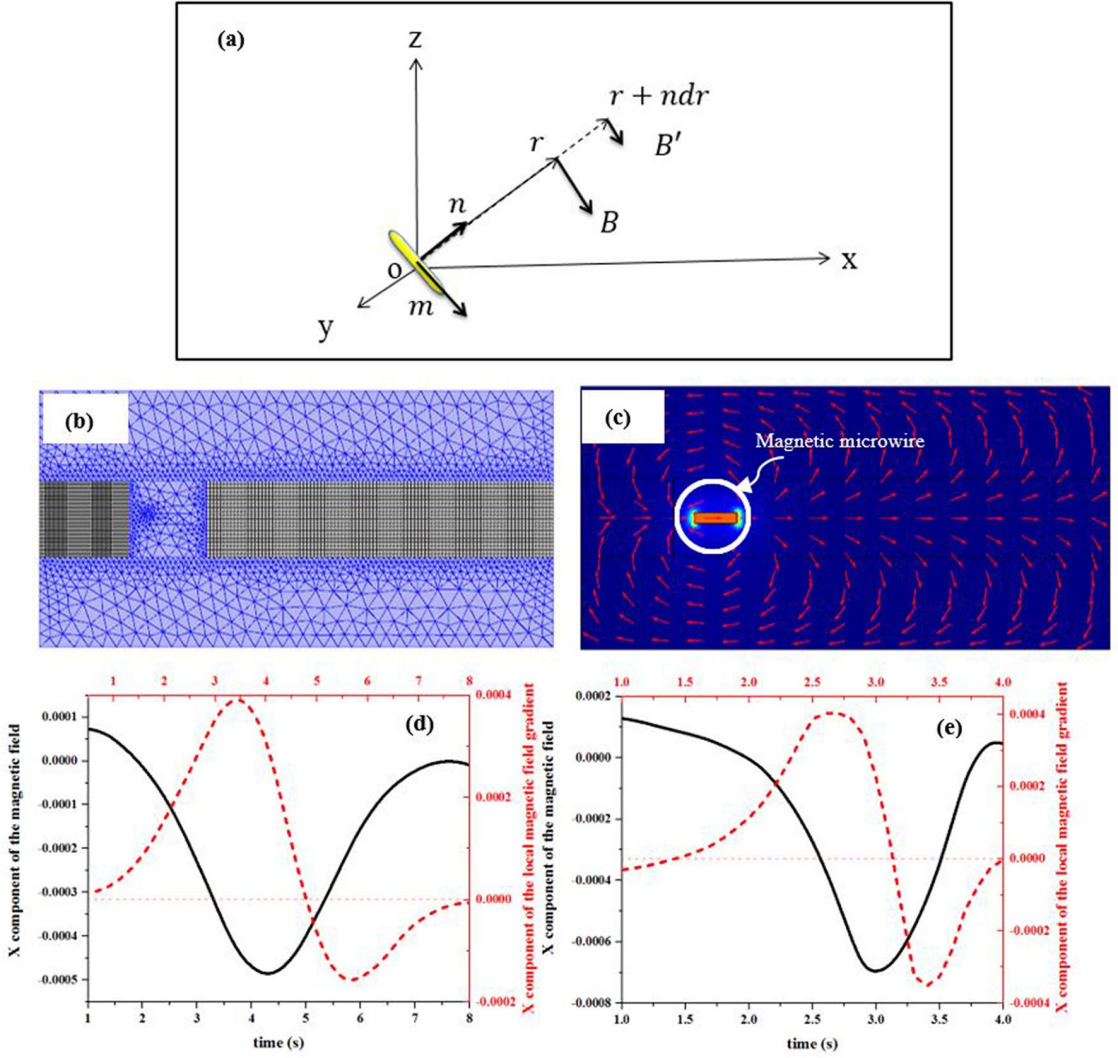
(**a**) The location of the magnetic dipole of $\overrightarrow{\mathrm{m}}$ relative to the atomic magnetometer. (**b**) Moving and mapped mesh for stationary and linearly translating domains, respectively, and (**c**) magnetic field lines created around the magnetic microwire. Simulation of the magnetic field and local magnetic gradient of the moving magnetic microwire with (**d**) constant and (**e**) variable velocity using COMSOL Multiphysics 5.4^53^.

As shown in Fig. 4a, $\mathrm{n}$ and $\mathrm{n}^{\prime}$ are common under a straight path away from the atomic magnetometer. Different change of the magnetic field between these two points can be obtained by using Eqs. (6) and (9),

10 $$\mathrm{dB}=-\frac{3}{\mathrm{r}}\mathrm{Bdr}.$$

By considering Eqs. (8) and (10), an equation for localization can be obtained as follows:

11 $$\mathrm{r}=-3{\mathrm{G}}^{-1}\mathrm{B}.$$

As shown, the position of the magnetic dipole can be obtained by measuring the magnetic field and local magnetic field gradient simultaneously^51,52^.

Consequently, the time derivative of the position r called “velocity” depends on the derivative of the magnetic field and local magnetic field gradient over time,

12 $$\mathrm{v}=\frac{\mathrm{dr}}{\mathrm{dt}}=-3\frac{\mathrm{d}}{\mathrm{dt}}\left({\mathrm{G}}^{-1}\mathrm{B}\right).$$

Time difference of these two parameters while the magnetic microwire experiences velocity variable can be modeled using the COMSOL Multiphysics 5.4. In the simulation, a moving magnetic microwire with a constant velocity of $\mathrm{v}$ and a moving magnetic microwire with an accelerated motion of $\mathrm{v}=\mathrm{at}$ were considered.

For a uniformly magnetized microwire, the induced magnetic field and local magnetic field gradient at any point around the microwire can be relatively estimated well based on simulation approaches. The simulation was performed by combining the deformed mesh interface with the Magnetic Fields, and Coefficient Form Boundary PDE interfaces. An x-oriented magnetic microwire similar to its real data was simulated. In addition, the microwire was described by using a ferromagnetic material.

For meshing this model, the moving section includes the magnetic microwire and a small air region around. Thus, linearly translating and stationary domains were considered as two different regions. To model the translational motion of the magnetic microwire in the linearly translating domain, the Moving Mesh interface was utilized. The other air stationary domains have a mapped mesh (Fig. 4b).

Now, to compute the deformation of the mesh, the boundaries adjacent to the translating domains (which are completely defined by translation function (x(t) and y(t))), those stationary domains with no deformation and some connecting boundaries were defined.

Further, the interface of Coefficient Form Boundary PDE was added to define these connecting lines. This interface defines the displacement along the boundaries by introducing new help variables.

The Magnetic Field (mf) interface is used to compute the magnetic field and local magnetic field gradient of the magnetic microwire. Then, a uniform background magnetic field was defined along the x axis in order to magnetize the microwire. In this background magnetic field, the microwire induced a magnetic field, the distribution of which is depicted in Fig. 4c for a specified time.

The black solid curves in Fig. 4d and e show the x component of magnetic field of the microwire with constant and variable velocity, respectively. The comparison of these curves demonstrates that the variable velocity can change the magnetic field over time and makes it asymmetric.

**Figure 5:**
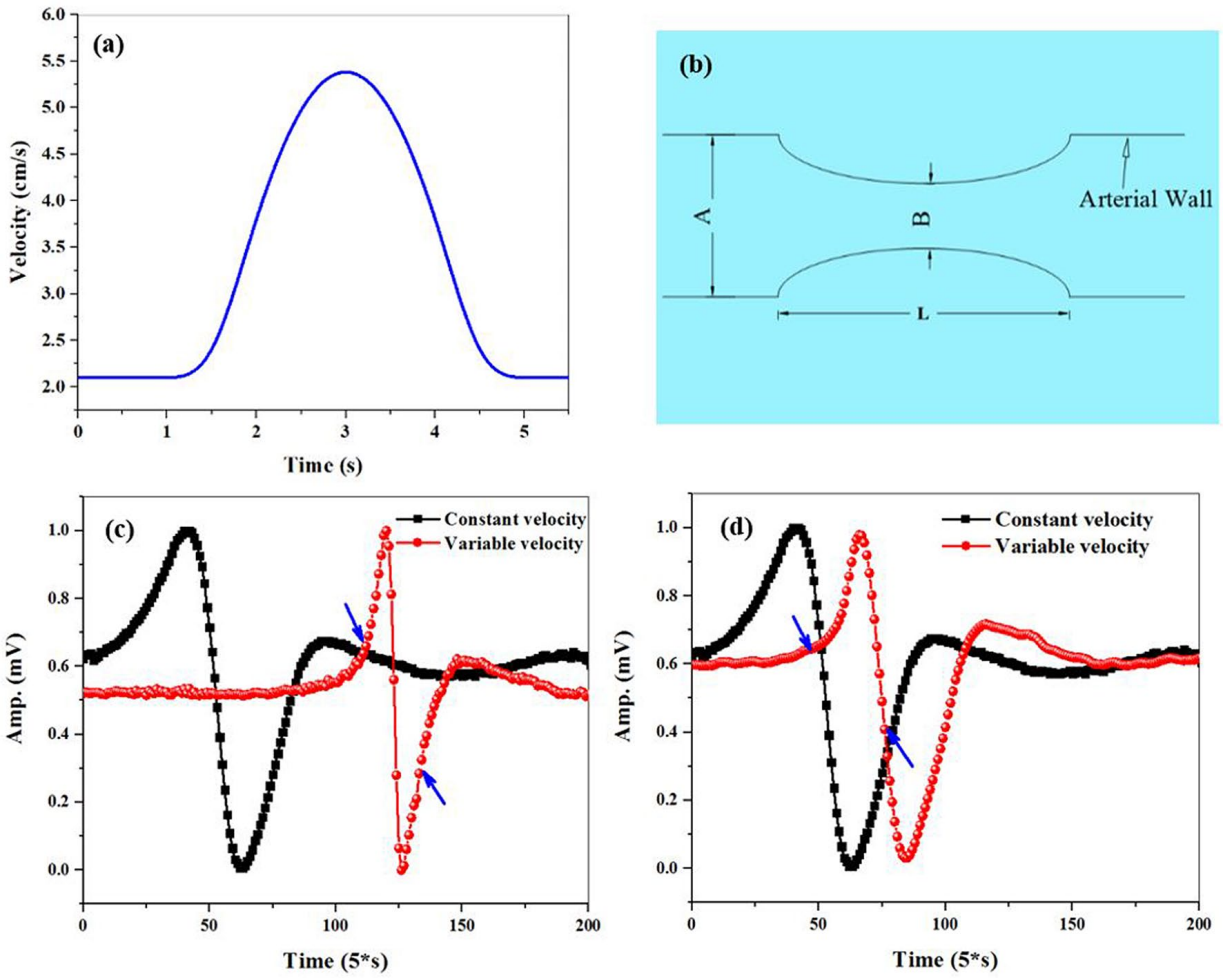
(**a**) A simple model scheme of a stenosis artery (A: diameter of the artery, B: diameter of the artery in the stenosis region, and L: length of the stenosis), and (**b**) sinusoidal variable velocity profile. The spike-like signals of the microwire for instantaneous velocity applied to the microwire when its location is (**c**) near the first edge of the vapor cell, and (**d**) near the middle of the vapor cell (time interval of applying instantaneous velocity marked with blue arrows).

Similarly, the red dashed curves in Fig. 4d and e are the X components of the local magnetic field gradient of the microwire moving with constant and variable velocity, respectively. By comparing these two curves, the local magnetic field gradient of the microwire shrinks through the time, and its shape goes out of symmetry in the case of variable velocity. These results confirm the fact that time derivative of the B and G depends on the instantaneous velocity of the magnetic microwire based on Eq. (12). This dependence is a logical justification for the experimental results obtained in Fig. 3c (the curve with red circles). To have a condition with the closest resemblance to the real situation of a stenosis artery, we considered a simple model scheme in Fig. 5a such as steady blood flow in a rigid stenosis tube. According to Poiseuille’s Law, the velocity of the flow along the tube depends on the area of the cross section. Thus, the velocity of the flow increases abruptly while decreasing the diameter of the blood vessel^18,54^.

Now, a sinusoidal variation of the instantaneous velocity was considered for modelling this velocity profile. Thus, the motor gearbox was driven with a sinusoidal voltage (Fig. 5b). The changes in the light intensity due to passing the microwire were detected as a spike-like signal. Figure 5c shows a comparison of two measured spike-like signals including the microwire with constant (black squares) and an abrupt change in the velocity (red circles). By applying a variation in instantaneous velocity, a slight change was observed in the spike-like signal width.

As shown in the spike-like signal in Fig. 5c, when the magnetic microwire location was near the first edge (second edge) of the Rb vapor cell, the use of the instantaneous velocity variations shrinks the width of the peak (or valley) over time. However, the width of the peak and valley decreases over time due to instantaneous velocity variations when the moving microwire passes the middle region of the Rb vapor cell (Fig. 5d).

This signal behavior in response to instantaneous velocity is in good agreement with simulation results in Fig. 4e. Based on the results, the effect of instantaneous velocity on the magnetic field and local magnetic field gradient of the moving magnetic microwire was confirmed. The simultaneous effect of these two parameters appears in the shape of the spike-like signal by which any abrupt change in velocity destroys symmetry. The location of the shape deformation changes according to the location of the stenosis relative to the Rb vapor cell.

### Formation of the spike-like signal in a stenosis artery phantom

The results obtained in the previous section persuade us that this system could be used to study instantaneous changes in blood velocity at stenosis regions in order to diagnose these lesions. For this purpose, an arterial phantom with two different lengths of stenosis (7 mm and 14 mm) were used. Droplets containing magnetic nanoparticles were passed through the phantom. The distance between magnetic droplets was designed in a way that separate signals were obtained for each droplet.

**Figure 6:**
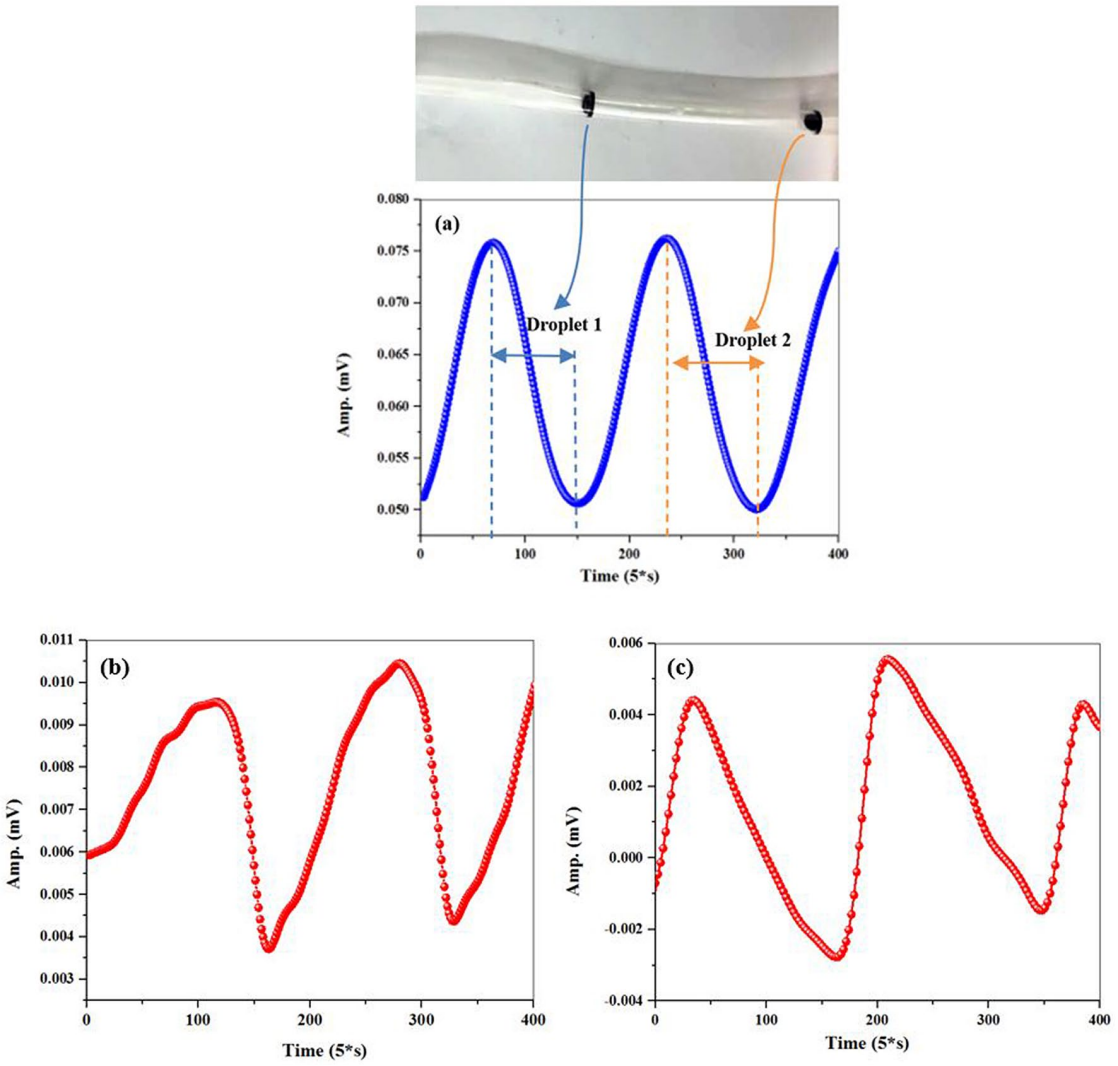
(**a**) The signal related to detection of two magnetic droplets through the arterial vessel phantom (**a**) without stenosis, (**b**) with a 7 mm stenosis, and (**c**) with a 14 mm stenosis.

Magnetic signal was measured before and after stenosis. Figure 6a shows the changes of the light intensity because of passing two magnetic droplets through a non-stenosis arterial phantom. Each time a magnetic droplet passes adjacent to the magnetometer, a symmetric spike-like signal with the same peak and valley shapes was detected. Then droplets containing the magnetic nanoparticles were passed through a stenosis tube. Figure 6b and c show a non-symmetric behavior in the spike-like signal which is in confirmation with the results obtained in the magnetic microwire detection. It can be concluded that the velocity of the magnetic fluid changes in the stenosis region. With respect to this point that the presence of stenosis in the vessels changes the velocity of the fluid, it can be concluded that the sensor is able to record the changes in blood flow velocity in the stenosis regions.

## Conclusion

Potential application of the atomic magnetometer in magnetic target detection demonstrates that it is capable of recording instantaneous velocity variations, along with the measurements of the position and velocity. Based on the results, the magnetic detection signal (named as the spike-like signal) was produced by simultaneous changes of the magnetic field and local magnetic field gradient of the moving target. A spike-like signal with a nearly symmetrical shape was obtained when the velocity of the target was constant. When an instantaneous variation was excreted in the microwire velocity, shrinkage was observed in the width of the signal. To confirm the results, the magnetic microwire was considered as a magnetic dipole. The simulation results of the moving magnetic dipole confirmed the fact that the time derivative of the magnetic field and magnetic field gradient relies on the instantaneous velocity variations of the magnetic microwire, which is in consistent with our experimental results. We also considered instantaneous velocity variations of the microwire similar to the actual state of the stenosis artery. Applying the instantaneous velocity variations shrinks the width of the peak (or valley) over time and the shape of this shrinkage depends on the location of the magnetic tracer relative to the magnetometer. Finally, passage of magnetic droplets through arterial phantom with two different lengths of stenosis have been studied. The obtained non-symmetric spike-like signal confirmed the great potential of the atomic magnetometer in detection of velocity changes of the fluid due to presence of stenosis in the vessels.
